# An organoid model derived from human adipose stem/progenitor cells to study adipose tissue physiology

**DOI:** 10.1080/21623945.2022.2044601

**Published:** 2022-03-17

**Authors:** Markus Mandl, Hans P. Viertler, Florian M. Hatzmann, Camille Brucker, Sonja Großmann, Petra Waldegger, Tina Rauchenwald, Monika Mattesich, Marit Zwierzina, Gerhard Pierer, Werner Zwerschke

**Affiliations:** a Division of Cell Metabolism and Differentiation Research, Research Institute for Biomedical Aging Research, University of Innsbruck, Austria; b Department of Plastic, Reconstructive and Aesthetic Surgery, Medical University of Innsbruck, Innsbruck, Austria

**Keywords:** Adipogenesis, adipocyte, adipose tissue, ageing, obesity, organoid, regenerative medicine, spheroid, stem cells

## Abstract

We established a functional adipose organoid model system for human adipose stem/progenitor cells (ASCs) isolated from white adipose tissue (WAT). ASCs were forced to self-aggregate by a hanging-drop technique. Afterwards, spheroids were transferred into agar-coated cell culture dishes to avoid plastic-adherence and dis-aggregation. Adipocyte differentiation was induced by an adipogenic hormone cocktail. Morphometric analysis revealed a significant increase in organoid size in the course of adipogenesis until d 18. Whole mount staining of organoids using specific lipophilic dyes showed large multi- and unilocular fat deposits in differentiated cells indicating highly efficient differentiation of ASCs into mature adipocytes. Moreover, we found a strong induction of the expression of key adipogenesis and adipocyte markers (CCAAT/enhancer-binding protein (C/EBP) β, peroxisome proliferator-activated receptor (PPAR) γ, fatty acid-binding protein 4 (FABP4), adiponectin) during adipose organoid formation. Secreted adiponectin was detected in the cell culture supernatant, underscoring the physiological relevance of mature adipocytes in the organoid model. Moreover, colony formation assays of collagenase-digested organoids revealed the maintenance of a significant fraction of ASCs within newly formed organoids. In conclusion, we provide a reliable and highly efficient WAT organoid model, which enables accurate analysis of cellular and molecular markers of adipogenic differentiation and adipocyte physiology.

## Introduction

Adipose stem/progenitor cells (ASCs) are crucial for adipose tissue (AT) homoeostasis, regeneration and expansion [1]. Their proper function is important for metabolic health and declines in obesity and ageing [2,3]. ASCs are also paramount in regenerative medicine and tissue engineering [4,5]. So far, these cells have been mainly studied in two-dimensional (2D) cell culture and mouse models to better understand self-renewal and adipocyte differentiation of the adipose lineage [2,6]. Adipogenic differentiation, also referred to as adipogenesis, is a highly orchestrated process subdivided into commitment of ASCs to pre-adipocytes and terminal differentiation [1,6,7]. Adipogenesis is governed by systemic factors, for example insulin, and signals derived from the adipose stem cell niche. The latter includes soluble factors such as mitogens and growth factors and insoluble components such as adhesion molecules on adipocytes and other niche cell surfaces and elements of the extracellular matrix (ECM) [8]. The composition and stiffness of niche cells and the ECM contribute to adipogenic regulation [1]. In 2D culture, high cell density and simultaneous exposure of ASCs to a defined adipogenic differentiation medium initiates the expression of a cascade of transcription factors, most important CCAAT/enhancer-binding protein (C/EBP) β and its target peroxisome proliferator-activated receptor (PPAR) γ 2, the master regulator of adipogenesis [6]. This leads to generation of adipocytes, the predominant cell type in white AT (WAT), which stores excess energy in form of triglycerides. Mature white adipocytes are morphologically characterized by an unilocular lipid droplet, express specific markers such as Fatty acid-binding protein 4 (*FABP4*) and secrete adipokines, for example adiponectin (*ADIPOQ*) or leptin (*LEP*), that are involved in the regulation of insulin sensitivity and satiety, defining the role of adipocytes as endocrine cells [1].

Three-dimensional (3D) cell culture techniques can be used to develop adipose spheroids derived from ASCs, which are capable to differentiate and self-organize into adipose organ-like structures [9,10]. In general, organoids grown from stem cells display a well-defined 3D geometry and various physiological aspects of intact tissues and organs [11]. In 3D culture, cell shape, stiffness and ECM, cell–cell and cell–matrix interaction and the physiological microenvironment more closely resemble intact tissues and influence gene expression and biological behaviour of the cells [12]. Although 3D cultures can probably not completely replace mechanistic studies in animal models, human organoids provide an alternative to animal experiments in accordance with the 3 R (replace, reduce, refine) principle and are promising experimental models for bridging the gap between *in vitro* and *in vivo* studies and animal models and humans [11]. Adipose organoids can be regarded as advanced models to study adipose tissue development and physiology [9]. In translational research, adipose organoids can be employed in tissue engineering, as building-blocks in autologous AT grafting and for preclinical drug discovery [5]. Although many cell types can secrete ECM components and self-assemble into aggregates, most ASC-derived adipose spheroid models employ scaffold/matrix-based systems [9^,^^10^,^13–15^], whereas scaffold-free 3D organoid models are less studied [13,16].

In the present study, we established a human ASC-derived organoid model recapitulating major features of WAT including aggregation, self-organization and adipocyte differentiation, as shown by highly efficient triglyceride formation and adiponectin secretion. Our protocol enables the quantitative analysis of adipogenic differentiation on the molecular level in organoids and will provide a cheap and useful tool for the study of adipose tissue biology and fat grafting in 3D models.

## Results

### Self-aggregated ASCs differentiate into adipose organoids with large adipocytes

**Figure 1.**
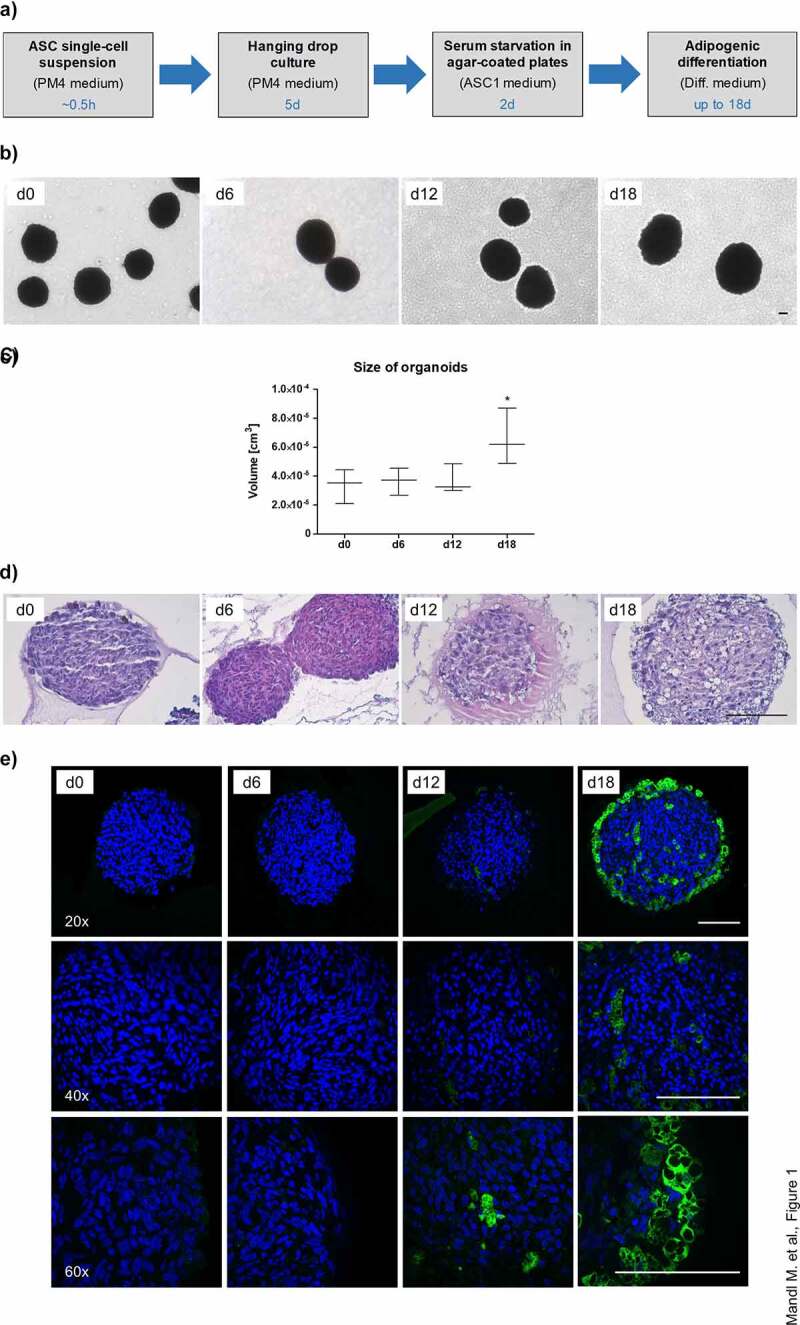
Generation of WAT organoids. (a) Overview of experimental procedures. Culture medium and the appropriate time scale are indicated. (b) Microphotographs of organoids during adipogenic differentiation in agar-coated 6-well plates. Magnification: 50×; Scale bar: 100 µm. A representative result of *n* = 3 different donors is shown. (c) Organoid size during adipogenesis. The diameter of *n* = 6–10 individual organoids per donor were measured using microphotographs and the volume was calculated as described in the Methods section. The data were pooled and values are presented as mean ± SEM of *n* = 3 different donors. Statistical analysis was done using one-way ANOVA with Dunnett's Multiple Comparison Test. (d) Haematoxylin/Eosin (H/E) staining of formalin-fixed paraffin-embedded (FFPE) organoids during adipogenesis. Sections: 4 µm; Magnification: 100×; Scale bar: 100 µm; A representative result of *n* = 3 different donors is shown. (e) Immunohistochemistry of FFPE organoids using an antibody against the adipocyte marker FABP4 (green). Nuclei were stained with DAPI (blue). Images were taken with the Confocal Scanner System Cell Voyager CV1000 (Yokogawa). A representative result of *n* = 3 donors is shown. Magnification as indicated. Scale bar: 100 µm.

**Figure 2.**
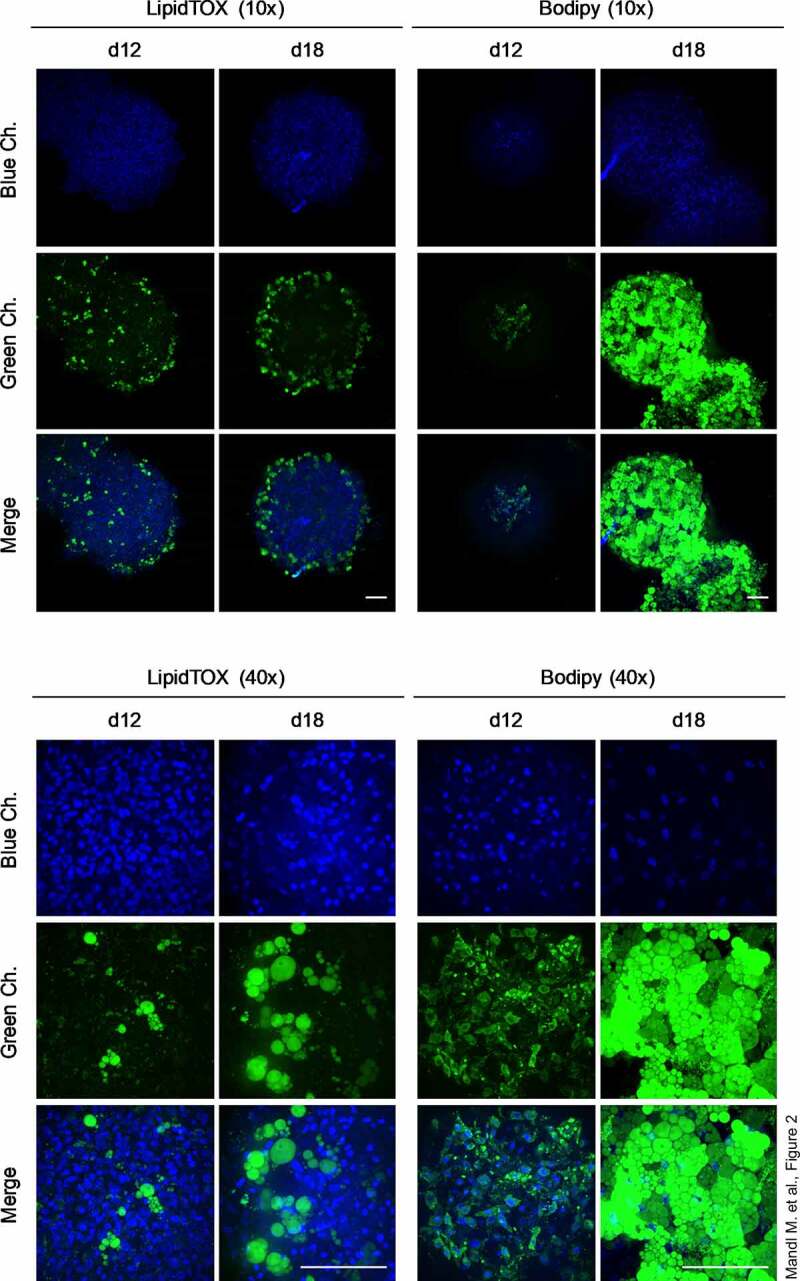
Intracellular triglyceride accumulation in organoids. Whole-mount staining of organoids employing the lipid-specific dyes LipidTOX™ and Bodipy™ on d 12 and d 18 of adipogenesis. Images were taken with the Confocal Scanner System Cell Voyager CV1000 (Yokogawa). A representative result of *n* = 3 different donors is shown. Magnification as indicated. Scale bar: 100 µm.

To establish a reliable and cost-efficient organoid system for the study of adipogenic differentiation and adipocyte physiology, we self-aggregated freshly isolated ASCs from subcutaneous (s)WAT of several human donors (Table 1) using a hanging drop cell culture technique. Afterwards, the cell aggregates were cultured on agarose-coated cell culture dishes (Figure 1a). Adipogenesis was stimulated by hormone cocktail and cell aggregates showed a significant increase in size after 18 d (Figure 1b and 1c). As demonstrated in Figure 1d, the spheroids were composed of a compact cell mass on d 0 and contained large rounded cells from d 12 on. Immunohistochemistry using an antibody against the adipocyte marker FABP4 identified these cells as adipocytes (Figure 1e). Adipocyte size varied among donors ranging from ~4.9 to ~14.4 µm in diameter as determined by the Adiposoft software applied on Haematoxylin/Eosin (H/E) stained sections of d 18 spheroids **(Supplementary Figure S1)**. Whole mount stainings with the lipid-specific probes LipidTOX™ and BODIPY™FLC16 confirmed intracellular lipid accumulation and the capability of adipocytes to uptake free fatty acids, respectively (Figure 2). In fact, the majority of cells in d 18 spheroids were adipocytes with large, pauci- and unilocular fat deposits (Figure 2). Cell viability was preserved during cell aggregation in hanging drops **(Supplementary Figure S2A)** and on the agarose-coated cell culture dishes in the course of the formation of adipose organoids **(Supplementary Figure S2B)**. Hence, our results demonstrate the capability of ASCs to form 3D structures and to self-organize via differentiation into adipose organ-specific adipocytes using the hanging drop/agarose-coated cell culture dish system.

**Table 1.** Donor characteristics

Donor	Sex	Age [y]	BMI [kg/m^2^]
1	f	21	25.04
2	f	28	24.22
3	f	28	21.6
4	f	28	22.86
5	f	34	25.95
6	f	35	22.23
7	f	37	23.05
8	f	50	27.99
9	f	61	26.11

Human sWAT samples were obtained from the lower abdomen of donors undergoing routine abdominoplasty at the Department of Plastic, Reconstructive and Aesthetic Surgery at the Medical University of Innsbruck, Austria. BMI: Body mass index; f = female, m = male, n. a. = not available.

To determine whether cell division of ASCs contributes to the increase in adipose organoid size, the expression of the proliferation marker KI67, which is expressed in all phases of the cell cycle except G0 [17], was analysed by immunohistochemistry. As shown in **Supplementary Figure S3A**, no KI67 positive cells were observed in the course of the generation of adipose organoids as judged by the absence of a specific nuclear KI67 staining. Moreover, expression of the S phase marker Cyclin A2 (*CCNA2*) [18] was not induced at the early time points investigated and remained hardly detectable during the formation of the adipose organoids **(Supplementary Figure S3B)**. These findings suggest that organoid enlargement is not caused by ASC proliferation but rather a consequence of adipogenic differentiation and increase in adipocyte size.

### Expression of adipogenesis and adipocyte marker in the course of adipose organoid genesis

**Figure 3.**
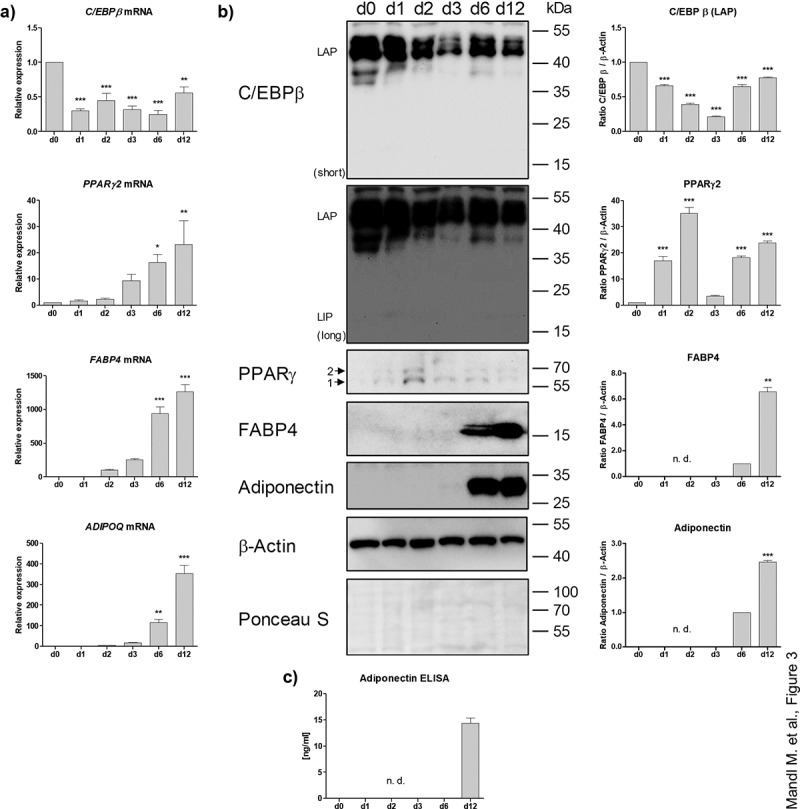
Analysis of adipogenic markers in the course of differentiation. (a) mRNA expression of adipogenic genes measured by RT-qPCR. A representative result of *n* = 3 different donors is shown. Values are presented as mean ±SEM of three technical replicates. Statistical analysis was done using one-way ANOVA with Dunnett's Multiple Comparison Test. (b) Western blot analysis of adipogenic markers corresponding to (a). Left panel: A representative result of *n* = 3 different donors is shown. β-Actin served as loading control. Right panel: Densitometric analysis. Values are presented as mean ± SEM of three measurements. Statistical analysis was done using one-way ANOVA with Dunnett's Multiple Comparison Test. (c) Adiponectin ELISA of cell culture supernatants corresponding to (a) and (b). n. d.: not determined.

To monitor the genesis of the adipose organoids, we quantified the expression of adipogenesis marker genes. Similar to 2D cell culture models of human ASCs [19], the early adipogenic regulator C/EBPβ was highly expressed at the mRNA (d 0: C_T_ ~24.8) and protein level in ASC aggregates before the onset of adipogenesis (Figure 3a and 3b). After the induction of adipogenesis C/EBPβ decreased to some extend but increased again from d 3 on. Similar to the human ASC 2D cell culture model [19], the ratio between the pro-adipogenic C/EBPβ LAP (LAP and full LAP) and the inhibitory C/EBPβ LIP isoforms was particularly high. The transiently induced expression of C/EBPβ (full LAP, LAP and LIP) early in adipogenesis in the human ASC 2D cell culture model [19] is not visible in the adipose organoid model, which showed very strong adipogenic differentiation and formation of mature adipocytes as indicated by the distinct expression of the adipogenic master regulator PPARγ2 and very strong induction of the adipocyte marker FABP4 and Adiponectin (*ADIPOQ*) (Figure 3a and 3b). Furthermore, leptin (*LEP*) mRNA expression, which is strongly regulated in proportion to adipocyte volume [20,21], was hardly detectable overall and tended to increase in differentiated organoids **(Supplementary Figure S4A)**. To finalize our molecular characterization, we tested whether organoids might contain SVF-derived endothelial cells (EC) or if EC-like cells might be generated by differentiation as shown in other models [22]. Low expression of EC marker genes (i.e. *CD31* and *VEGFR2* [22]) on d 0 and d 12, despite a transient regulation, suggested the absence of a stable major EC fraction within WAT organoids **(Supplementary Figure S4B)**.

The physiological importance of the adipose organoids is underscored by the high adiponectin secretion on d 12 (Figure 3c) and their lipolytic capacity, as demonstrated by hormone-sensitive lipase (HSL) activation by Ser660 phosphorylation [23] and glycerol release **(Supplementary Figure S5)**. These data confirm the differentiation of ASCs into functional adipocytes and provide a reliable model system to study adipogenesis and adipocyte physiology in human adipose organoids.

### Adipose organoids retain a quiescent stem-cell population

**Figure 4.**
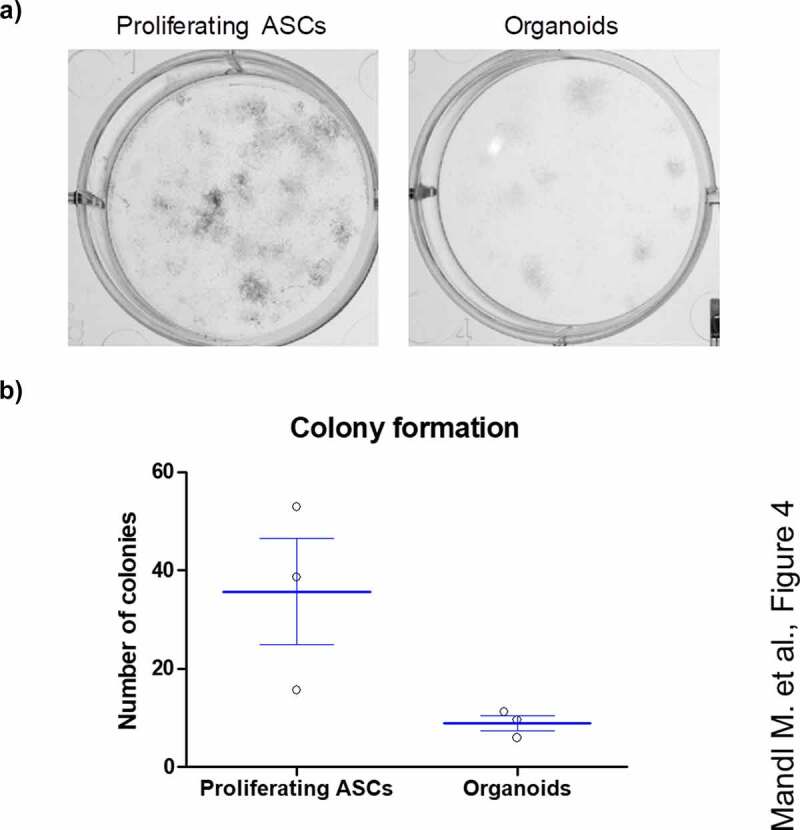
ASCs derived from adipose organoids retain the capability to form colonies. (a) Colony formation assays by ASCs derived from collagenase I-digested organoids and proliferating ASCs directly isolated from WAT of given donors are shown. Colonies are stained with crystal violet and counted. A representative result of *n* = 3 different donors, mean ± SEM, is shown. (b) Number of colonies formed by SVF-derived proliferating ASCs and digested organoids among *n* = 3 different donors. Three wells or organoids were used for each condition and donor.

To test whether the adipose organoids harbour a functional stem-cell population, organoids derived from ASCs from three different donors were harvested on d 18, disintegrated with collagenase I to obtain single-cell suspensions and colony formation assays were conducted. Proliferating ASCs derived from donor-matched SVFs were used for comparison. As shown in Figure 4, the capability to form distinct colonies was preserved in adipose organoid-derived cells. In agreement with the absence of proliferating cells in the adipose organoids **(Supplementary Figure S3)**, the colony formation assays suggest the presence and maintenance of a quiescent stem cell population within adipose organoids.

## Discussion

For the study of adipose tissue biology, several models exist, none of which mimics human physiology completely [11,13]. In the present study, we established an easy, reliable and cheap adipose organoid model using freshly isolated human ASCs and a scaffold-free system based on the hanging drop technique for generation of spheroids and subsequent cultivation of spheroids on agarose-coated cell culture dishes. Technical considerations (e.g. culture conditions, induction of adipogenesis, time points) were based on our previous studies demonstrating the successful differentiation of human ASCs into adipocytes [24–28]. However, we show that this approach leads to robust development of physiological functional adipose organoids.

Our study underscores that freshly isolated human ASCs support attachment and aggregation in hanging drop cultures [29] to generate 3D cellular structures that enhance adipogenic differentiation and the development of mature adipocytes. We employed bacteriological-grade petri dishes for hanging drop formation and ASC self-aggregation similar to previous work [16]. During further cultivation, the prevention of plastic-adherence of ASCs is critical for organoid generation and maintenance avoiding both organoid fusion and dis-aggregation. A succesful generation of adipose organoids was also shown in other studies [30]. Turner *et al*. [31] used specifically coated tissue culture plates to prevent the attachment of human ASCs leading to aggregation [31]. Al-Ghadban et al. [32] generated adipose spheroids without upstream hanging drops seeding ASCs directly on agarose-coated cell culture dishes on rotating orbital shakers to facilitate ASC accumulation and spheroid formation. Others used the hanging drop technique in combination with subsequent cultivation on low-attachment plates for the generation of adipose spheroids from immortalized pre-adipocyte cell lines and primary stromal vascular fraction (SVF) cells [16] or employed a levitation tissue culture system to generate adipose organoids derived from the murine SVF [13].

Organoids generated by our protocol reflect physiological characteristics of WAT such as self-organization, differentiation of precursor cells and generation of adipocytes accumulating large multi- and unilocular fat droplets. Adipocyte size within organoids varied from ~4.9 to ~14.4 µm in diameter, which is in agreement with the variable dimensions of human white adipocytes although adipocytes in human WAT can reach larger dimensions (diameter ≤20 up to 300 µm) [33]. Consistently, a robust expression of the pro-adipogenic key regulators C/EBPβ and PPARγ2 and adipocyte marker (*FABP4, ADIPOQ*) was detected. The physiological function of adipocytes was confirmed by their capability to accumulate lipids in large droplets and to secrete adiponectin, a major adipokine [34]. These data suggest that our adipose organoid model can be used to study adipogenesis, adipocyte maturation and expansion.

In general, different cell layers can be distinguished within an organoid or related cellular structures, a necrotic core, a zone of quiescent cells, proliferating and/or differentiating compartments [35,36]. The thickness of these areas is mainly governed by organoid size reflecting the availability of oxygen and nutrients as provided by diffusion. In addition, metabolic waste accumulating within an organoid plays a critical role [35,36]. Based on these considerations, we used an initial number of 20,000 ASCs to generate adipose organoids with a reasonable diameter. In fact, the radius of our spheroids was between 150 and 200 µm, which corresponds to the oxygen diffusion minimum that guarantees cell survival [37]. Thus, as expected, we obtained neither morphological nor biochemical signs of cell death inside the organoids and cell viability was maintained throughout adipogenesis.

We detected no cell proliferation within the adipose organoids, but colony formation assays of collagenase I-digested organoids on d 18 after the induction of adipogenesis revealed the existence of a quiescent stem cell population. These findings are in agreement with a recent report by Al-Ghadban et al. [32].

In the present study, we used sWAT samples derived from nine formerly obese donors undergoing elective plastic abdominal surgery after weight loss. All donors were females. The influence of sex was not considered. Whether organoid formation is altered when ASCs from obese donors or other AT depots are used, remains to be addressed in future studies.

In conclusion, we established a reliable, scaffold-free, cost-efficient, and quantifiable human adipose organoid model to study ASC and adipocyte functions in a recapitulated physiological microenvironment, which will be a useful tool in future studies on adipose tissue biology.

## Materials and methods

### White adipose tissue samples and donor characteristics

Subcutaneous white adipose tissue (sWAT) samples were obtained from patients undergoing elective body-contouring surgery at the Department of Plastic, Reconstructive and Aesthetic Surgery at the Medical University of Innsbruck, Tyrol, Austria. All patients gave their informed written consent. The study was approved by the Ethics Committee of the Medical University of Innsbruck (Austria) according to the Declaration of Helsinki. sWAT samples were taken from the lower abdomen of the subcutaneous fat layer between the rectus sheath and the fascia of scarpa. None of the patients suffered from malignant or severe metabolic diseases. In total, sWAT specimens derived from *n* = 9 donors were used. Detailed characteristics and clinical parameters are provided in Table 1.

### Isolation and cultivation of human adipose stem/progenitor cells (ASCs)

All isolation steps were carried out under sterile conditions using an appropriate laminar flow (Biosafety Level 2). sWAT samples were rinsed twice with PBS and dissected. Blood vessels and connective tissue were removed. Subsequently, sWAT samples were cut into millimetre-sized pieces and the amount was measured. Next, Collagenase I digestion (200 U/ml Collagenase I (CLS Type I, Worthington Biochemical Corp., Lakewood, NJ) in PBS supplemented with 2% w/v BSA) was performed under stirring for 1 h at 37°C. For 1 mg of sWAT, 3 ml of digestion solution were used. The mixture was cleared by filtration using a sieve, transferred into 50 -ml tubes and centrifuged (10 min, 200×*g*, RT). The pellet was resuspended in 30 ml erythrocyte lysis buffer (0.155 M NH_4_CI, 5.7 mM K_2_HPO_4_, 0.1 mM EDTA, pH 7.3) and incubated for 10 min at RT. Next, the solution was filtered using a cell-strainer (pore size 100 µm) and centrifuged (10 min, 200×*g*, RT). The cell pellet, the stromal vascular fraction (SVF), was re-suspended in 10 ml ASC2 medium (DMEM/F-12 medium with HEPES and L-Glutamine (Gibco, Vienna, Austria, #31,330,095), supplemented with 33 μM Biotin, 17 μM Pantothenate, 20 μg/ml Ciprofloxacin and 10% FCS (Gibco, Vienna, Austria)), filtered through a cell-strainer (pore size 35 µm) and counted with a Neubauer chamber. SVF cells were seeded into six-well plates at a density of ~2 × 10^5^ cells/cm^2^ and allowed to attach for 20 h using canonical cell culture conditions (37°C, 5% v/v CO_2_, humidified atmosphere). Subsequently, the medium was replaced by serum-free ASC1 medium (as described for ASC2 medium but without FCS). Cells were cultured in ASC1 medium for 6 d and the medium was changed regularly every 2–3 d. ASCs were harvested by trypsinization and seeded into 175 cm^2^ cell culture flasks at a density of 5000–8000 cells/cm^2^ using ASC2 medium (defined as passage −1). On the next day, the supernatant was replaced by PM4 medium (ASC1 medium supplemented with 2.5% FCS, 10 ng/ml EGF, 1 ng/ml bFGF, 500 ng/ml Insulin). The medium was exchanged regularly every 2–3 d and ASCs were sub-cultured at ~70% confluence in a ratio of 1:2 using ASC2 medium. On the next day, the medium was again replaced by PM4. For all experiments, only ASCs up to passage 6 were used.

### Generation of organoids

ASCs were amplified in PM4 medium on cell culture dishes, harvested by trypsinization, counted and suspended in PM4 medium to obtain a final cell density of 1 × 10^6^ cells/ml. Self-aggregation of ASCs was achieved by employing a hanging drop cell culture technique. Therefore, 20 µl droplets of the cell suspension were put onto the inner side of a lid of bacteriological-grade petri dishes (diameter 10 cm), inverted and placed onto dishes filled with 20 ml sterile PBS to avoid evaporation. Hanging drops were cultured for 5 d without medium changes at standard cell culture conditions (37°C, 5% CO_2_, humidified atmosphere). Afterwards, hanging drops were counted and transferred into a 50 ml Falcon tube using a sterile blue pipette tip cut off at the top by thorough rinsing with PBS. Cell aggregates were centrifuged (300×*g*, 1 min) and carefully suspended in serum-free ASC1 medium to obtain a concentration of 10–14 spheroids/ml. Next, spheroids were seeded in a total volume of 1 ml ASC1 medium in agar-coated 6-well plates (2% w/v Agar in PBS; 1.5 ml/well) to prevent plastic adhesion and incubated for 2 d (defined as d 0). Adipogenesis was initiated according to our well-established protocol as described previously [24]. Briefly, spheroids were treated with differentiation medium (ASC1 medium supplemented with 0.2 µM insulin, 0.25 µM dexamethasone, 2.5% FCS, 10 µg/ml transferrin and 0.5 mM 3-Isobutyl-1-methylxanthine (IBMX); DM; 1 ml/well) for 3 d to induce adipogenic differentiation followed by the incubation in DM without IBMX until d 18. The medium was exchanged every 3 d (between d 3 and d 18) using 1 ml of fresh DM without IBMX and the spheroids were grown without any shaking. Microphotographs were taken on d 0, d 6, d 12 and d 18 of adipogenesis using an inverse microscope (DMi1, Leica). The diameter of organoids was measured using ImageJ software (version 1.47, National Institutes of Health, USA) and the volume was calculated as described in Klingelhutz *et al*. [16]. General considerations regarding the number of organoids required for certain assays are provided in **Supplementary Table S3**.

### Whole mount staining

Organoids were transferred into a 1.5 -ml tube, centrifuged (1000 rpm; 30 s) and washed with PBS followed by whole mount staining without fixation in 500 µl PBS using specific probes as described below. Intracellular lipids were stained using LipidTOX™ (Invitrogen, #H34475) diluted 1:100 for 1 h at room temperature (RT). BODIPY™FLC16 (Invitrogen, #H34475; 2 mM in DMSO), which mimics fatty acid (FA) transport and metabolism in cells [38], was used to determine FA uptake in adipocytes and applied 1:100 for 1 h at RT. Live/death discrimination was achieved with Propidium iodide (PI; Sigma-Aldrich; #P4170; 1 mg/ml in PBS) diluted 1:100. Staining of cell nuclei was done with Hoechst (Sigma-Aldrich, #B2261; 1 mg/ml in PBS; diluted 1:50). Finally, organoids were washed with PBS and analysed immediately using a confocal microscope (Confocal Scanner System – Cell Voyager CV1000; Yokogawa).

### Histochemistry and immunohistochemistry

For histochemistry, organoids were transferred into a 15-ml tube, centrifuged (300×*g*; 2 min), washed with PBS and suspended in 150 µl citrated human plasma (Sigma-Aldrich; #P9523) followed by the addition of 150 µl Thrombin (1000 U/ml; Sigma-Aldrich; #T4648). After coagulation (occurring after ~10 min), samples were fixed in 4% PFA/PBS overnight at 4°C, dehydrated (70% EtOH: 2 × 15 min; 85% EtOH: 2 × 15 min; 95% EtOH: 2 × 15 min; 100% EtOH: 2 × 30 min; EtOH-xylene (1:1): 2 × 15 min; EtOH-xylene (1:2): 2 × 15 min; xylene: 2 × 15 min; Xylene (60°C): 1 × 15 min) and embedded into paraffin (at 60°C: xylene–paraplast (2:1): 1 × 30 min, xylene-paraplast (1:1): 1 × 30 min; xylene–pparaplast (1:2): 1 × 30 min; araplast: 2 × 30 min; paraplast: overnight; embedding). Sections (4 µm) were obtained using a microtome. Samples were deparaffinised, re-hydrated with a graded alcohol series (xylene: 2 × 10 min; 5 min each: 100% EtOH; 75% EtOH; 50% EtOH; 25% EtOH) and washed in TBS (2× 2 min). Representative sections were stained with haematoxylin/eosin (H/E; Roth).

For immunohistochemistry, antigen retrieval was done with 10 mM citrate buffer (pH 6.0) at 95°C for 15 min. Samples were chilled at room temperature for 30 min, washed twice with TBS for 2 min and treated with 5% H_2_O_2_ in 100% EtOH for 15 min. Unspecific binding sites were blocked with 1% BSA in 0.1% TBS-Tween supplemented with 10% normal goat serum for 30 min at RT. Primary antibodies **(Supplementary Table S1)** were diluted 1:100 in 1% BSA in 0.1% TBS-Tween and applied overnight at 4°C. After washing, samples were incubated with an appropriate FITC-conjugated secondary antibody (diluted 1:200; **Supplementary Table S1**) for 45 min at RT. To verify specificity, the primary antibody was omitted as negative control. Finally, samples were counterstained with DAPI and mounted.

### Measurement of adipocyte size

Microphotographs of H/E stained tissue sections of d 18 organoids were analysed using the Adiposoft software plugin [39] for ImageJ-win64 (Fiji; 1.53c, National Institutes of Health, USA) according to the protocol provided (https://imagej.net/plugins/adiposoft) [39]. Subsequently, the radius (*r*) and diameter (*d* = 2 *r*) of adipocytes were calculated from the area (*A*) measured by Adiposoft using the formula *A* = *r*^2^*π (i.e. *d* =$\sqrt[{2*}]{\frac{A}{\pi }}$) assuming a spherical cell shape [40–42].

### Flow cytometry

Spheroids were transferred into a 1.5-ml tube, washed with PBS and digested with 50 µl Collagenase I (200 U/ml in 2% BSA/PBS; Worthington Biochem.) for 1 h at 37°C. Cell viability was determined employing the Annexin V-FITC Apoptosis Detection Kit according to the manufacturer´s protocol and quantified on a LSRFortessa™ Flow Cytometer (BD Biosciences). Data analysis was done using FlowJo software (version 10.5.2; BD).

### Gene expression analysis

To gain RNA samples, organoids were transferred into a 1.5-ml tube, centrifuged and washed with PBS. Cells were lysed with 350 µl RLT buffer (RNeasy Plus Micro Kit; Qiagen) followed by homogenization using a pistil. RNA isolation and cDNA synthesis was done exactly as described for 2D cultures [27]. Gene expression was measured on a QuantStudio 7 Real-Time PCR system (Applied Biosystems) using SYBR green chemistry (AceQ qPCR SYBR Green, Vazyme Biotech). Primer sequences are provided in **Supplementary Table S2**. Expression levels were calculated using the comparative relative quantification (ΔΔC_T_) method and β-actin (*ACTB*) as endogenous control, which showed a comparable expression pattern among samples **(Supplementary Figure S6)**.

### Western blot analysis

To gain protein samples, organoids were transferred into a 1.-ml tube, centrifuged, washed with PBS and lysed in 80 µl SDS buffer. Homogenization was done using a pistil. Western blot analysis was carried out as previously described [27]. Antibodies are listed in **Supplementary Table S1**.

### Enzyme-linked immunosorbent assay (ELISA)

Adiponectin secreted into the cell culture supernatant, which was collected (1 ml) at indicated time points and at the same time when the medium was exchanged (every 3 d), was measured by ELISA (ELISA MAX™, Deluxe Set, Human Adiponectin; BioLegend) as described in the supplier´s protocol.

### Glycerol assay

Glycerol released by adipocytes into the culture medium was determined with the Glycerol Assay Kit (Sigma-Aldrich; #MAK117) according to the manufacturer’s instructions. The cell culture supernatant (1 ml) was collected at indicated time points and at the same time when the medium was exchanged (every 3 d).

### Colony formation assay

To obtain a single-cell suspension, spheroids were digested with 50 µl collagenase I (200 U/ml in 2% BSA/PBS; Worthington Biochem.) for 1 h at 37°C. Subsequently, cells were seeded in 6-well plates at a density of 1000 cells per well (~100 cells/cm^2^) and cultured until d 16. Fixation was carried out with Methanol: Acetone (1:1) for 5 min at RT. Colonies were stained with Crystal violet (0.2 mg/ml in 20% EtOH) and counted. The colony formation capacity of three individual spheroids per donor was compared to donor-matched proliferating ASCs measured in triplicates.

### Statistics

For statistical analysis, GraphPad Prism 5 software (GraphPad Software Inc., La Jolla, CA, USA) was used. Each experiment was done with a minimum of *n* = 3 biological replicates (i.e. donors). All measurements were done in triplicates. Values are given as mean ± standard error of the mean (SEM). Statistical comparison was achieved using the un/paired two-tailed *t*-test or ANOVA depending on the type of the data set and as mentioned in the corresponding figure legend. *p* Values ≤0.05 were considered to be significant and indicated as follows: * = *p* < 0.05; ** = *p* < 0.01; *** = *p* < 0.001.

## Supplementary Material

Supplemental Material

## Data Availability

The authors confirm that the data supporting the findings of this study are available within the article and its supplementary materials.

## Abbreviations

ADIPOQ Adiponectin

ASCs Adipogenic stem/progenitor cells

AT Adipose tissue

BMI Body mass index

C/EBPβCCAAT/enhancer-binding protein β

CCNA2Cyclin A2

ECM Extracellular matrix

ELISA Enzyme-linked immunosorbent assay

FABP4Fatty acid-binding protein

FFPE Formalin-fixed Paraffin embedded

H/E Haematoxylin/Eosin

PPARγPeroxisome proliferator-activated receptor γ

SVF Stromal vascular fraction

sWAT Subcutaneous white adipose tissue

WAT White adipose tissue
