# Supplementary figures and images for: An organoid model derived from human adipose stem/progenitor cells to study adipose tissue physiology
Source: Adipocyte. 2022 Mar 17;11(1):164–74. doi: 10.1080/21623945.2022.2044601 (PMC8932919; doi:10.1080/21623945.2022.2044601)

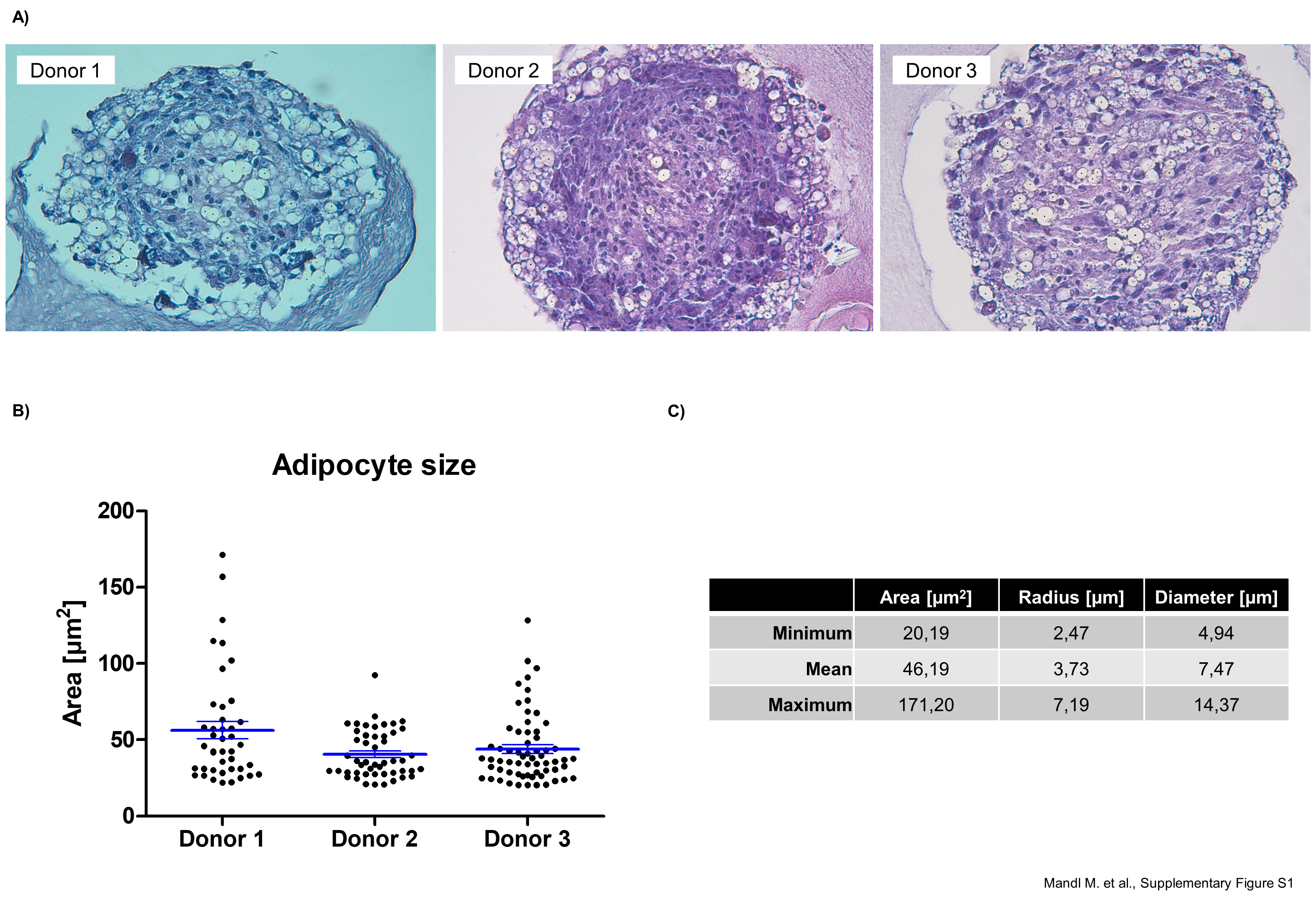

Supplement: Supplemental Material [file KADI_A_2044601_SM7004.zip › supplementary/Supplementary Figure S1 Adipocyte size.tif]

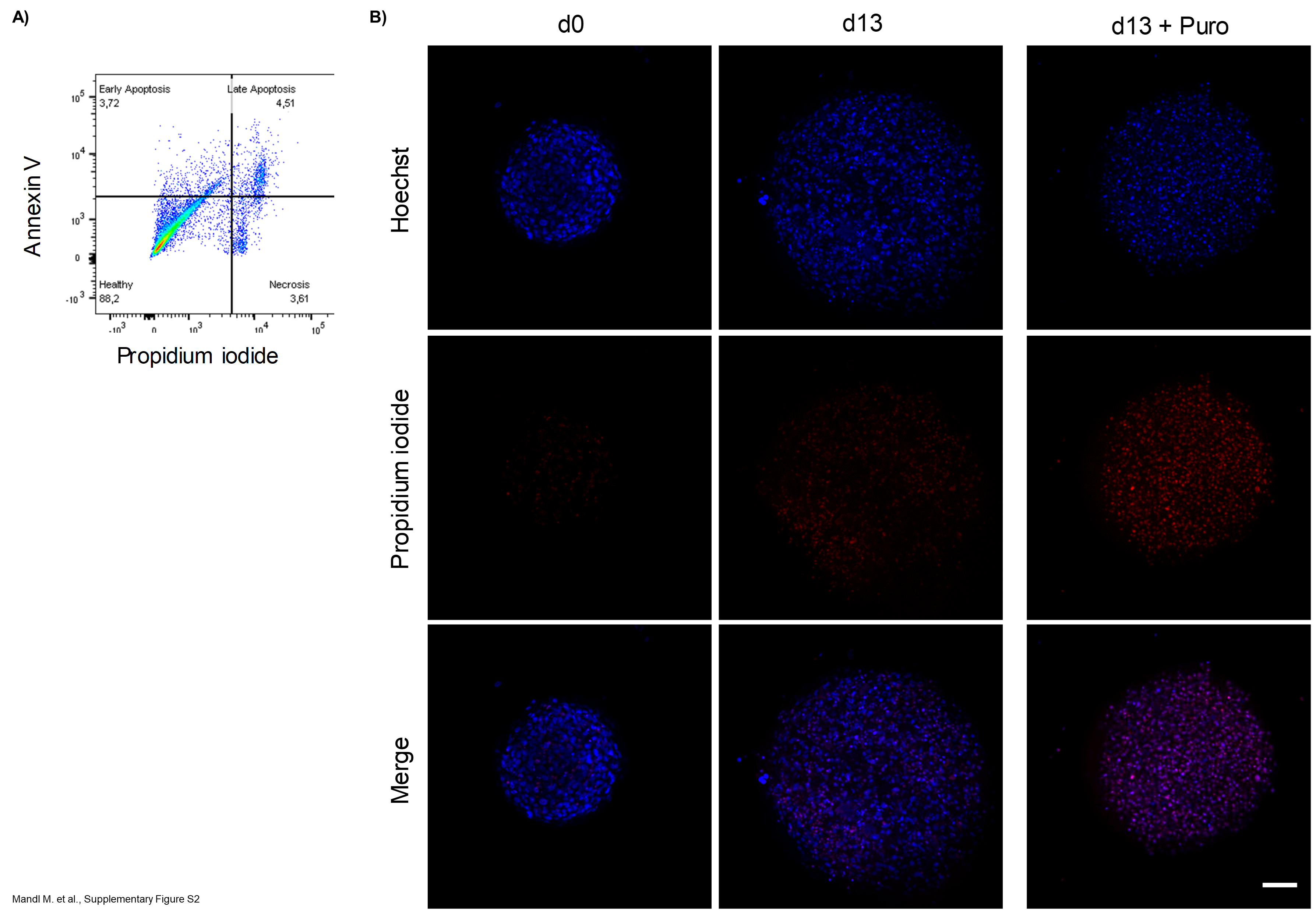

Supplement: Supplemental Material [file KADI_A_2044601_SM7004.zip › supplementary/Supplementary Figure S2 Viability.tif]

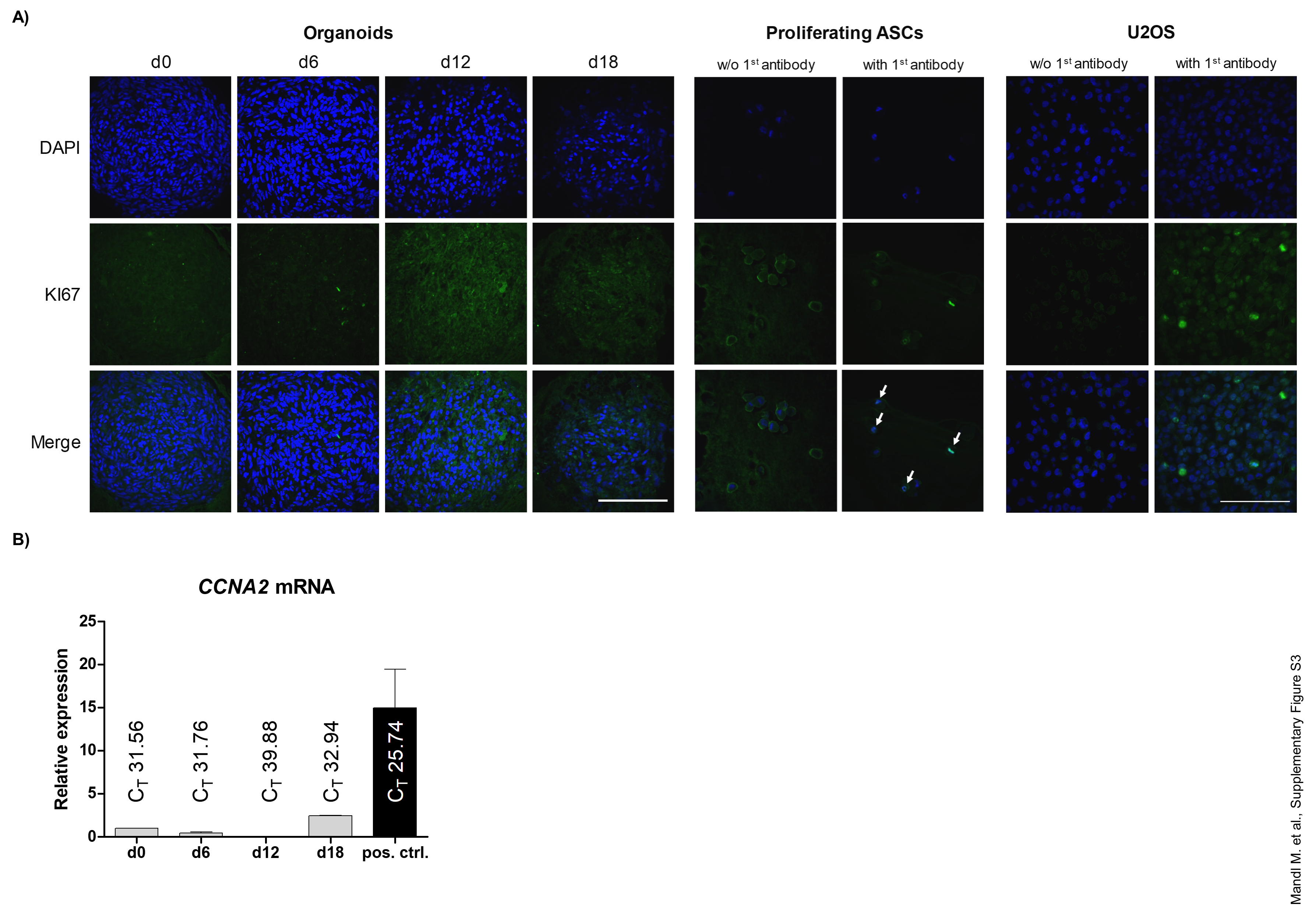

Supplement: Supplemental Material [file KADI_A_2044601_SM7004.zip › supplementary/Supplementary Figure S3 KI67 IHC.tif]

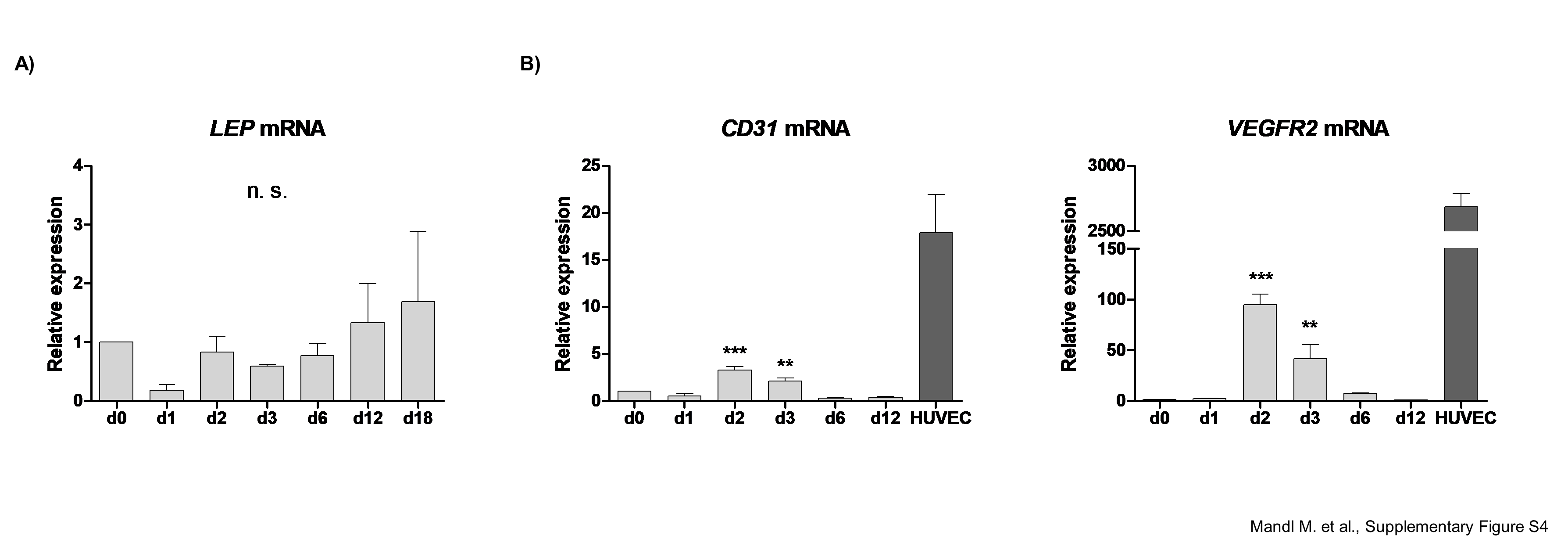

Supplement: Supplemental Material [file KADI_A_2044601_SM7004.zip › supplementary/Supplementary Figure S4 Leptin and EC marker expression 28012022.tif]

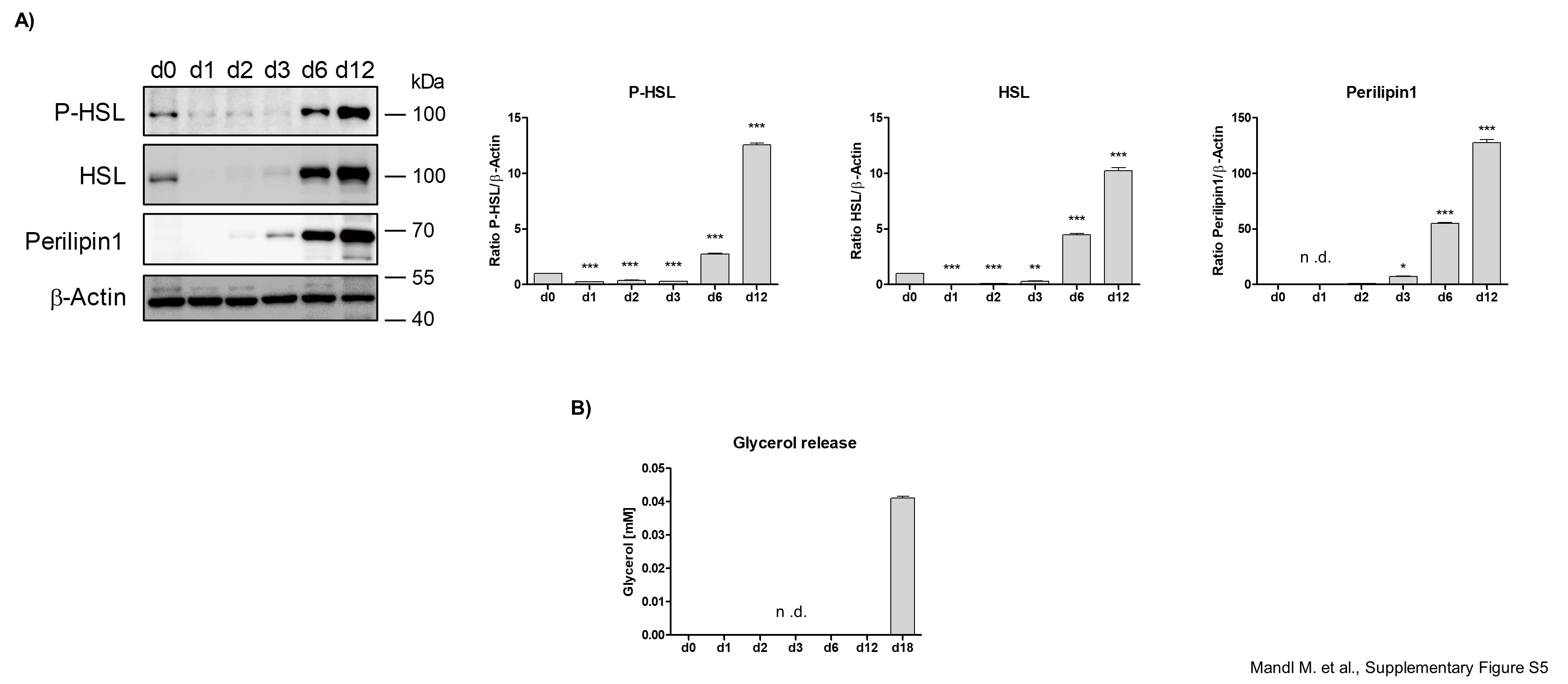

Supplement: Supplemental Material [file KADI_A_2044601_SM7004.zip › supplementary/Supplementary Figure S5 Lipolysis 28012022 (1).tif]

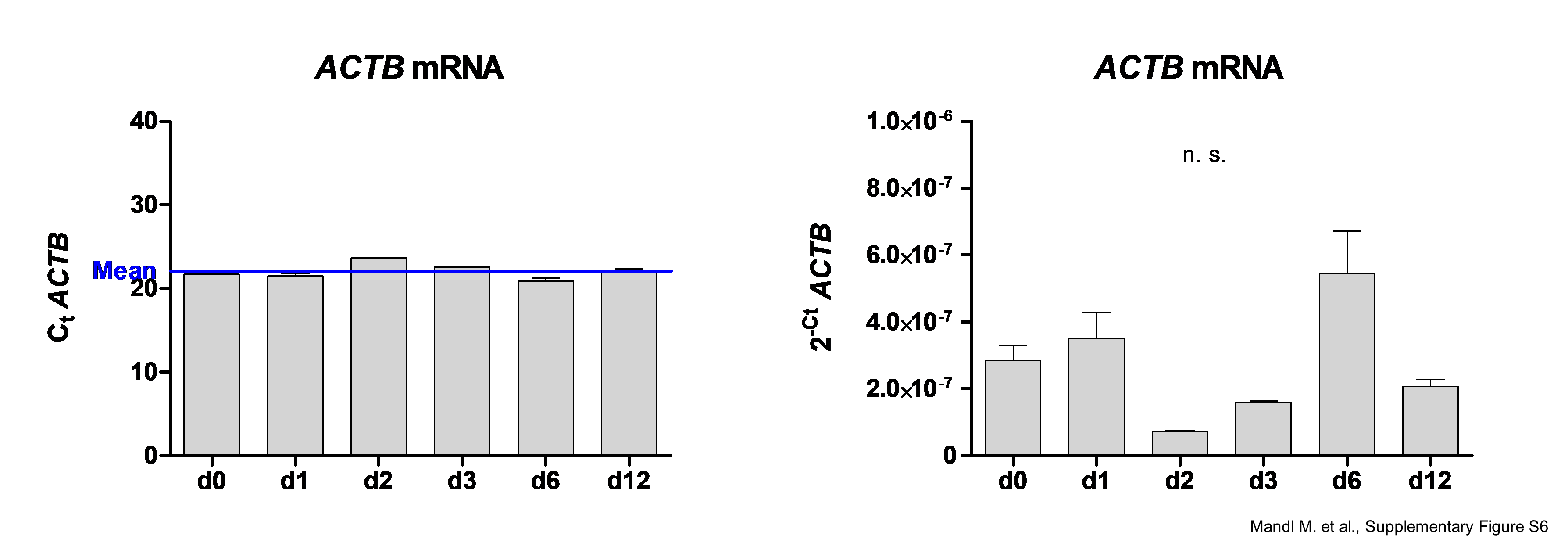

Supplement: Supplemental Material [file KADI_A_2044601_SM7004.zip › supplementary/Supplementary Figure S6.tif]
